# sc-ImmuCC: hierarchical annotation for immune cell types in single-cell RNA-seq

**DOI:** 10.3389/fimmu.2023.1223471

**Published:** 2023-07-20

**Authors:** Ying Jiang, Ziyi Chen, Na Han, Jingzhe Shang, Aiping Wu

**Affiliations:** State Key Laboratory of Common Mechanism Research for Major Diseases, Suzhou Institute of Systems Medicine, Chinese Academy of Medical Sciences & Peking Union Medical College, Suzhou, Jiangsu, China

**Keywords:** immune cell identification, scRNA-seq, hierarchical annotation, immune cell signature sets, ssGSEA

## Abstract

Accurately identifying immune cell types in single-cell RNA-sequencing (scRNA-Seq) data is critical to uncovering immune responses in health or disease conditions. However, the high heterogeneity and sparsity of scRNA-Seq data, as well as the similarity in gene expression among immune cell types, poses a great challenge for accurate identification of immune cell types in scRNA-Seq data. Here, we developed a tool named sc-ImmuCC for hierarchical annotation of immune cell types from scRNA-Seq data, based on the optimized gene sets and ssGSEA algorithm. sc-ImmuCC simulates the natural differentiation of immune cells, and the hierarchical annotation includes three layers, which can annotate nine major immune cell types and 29 cell subtypes. The test results showed its stable performance and strong consistency among different tissue datasets with average accuracy of 71-90%. In addition, the optimized gene sets and hierarchical annotation strategy could be applied to other methods to improve their annotation accuracy and the spectrum of annotated cell types and subtypes. We also applied sc-ImmuCC to a dataset composed of COVID-19, influenza, and healthy donors, and found that the proportion of monocytes in patients with COVID-19 and influenza was significantly higher than that in healthy people. The easy-to-use sc-ImmuCC tool provides a good way to comprehensively annotate immune cell types from scRNA-Seq data, and will also help study the immune mechanism underlying physiological and pathological conditions.

## Introduction

The infiltration and quantity of immune cells in tissues are closely related to the occurrence, treatment response, and development of diseases (1–3), such as cancer and inflammatory disease (4). Quantitatively identifying immune cells in tissues can provide new insights and methods for disease treatment and prevention. Traditional experimental methods, such as flow cytometry (5), affinity purification (6), and immunohistochemistry (7), are capable of qualitatively and quantitatively measuring immune cells. However, these methods are of limited use when the markers for some cell types are not clear, and they impractical in the case of large-scale samples due to their time consumption (8).

Computational strategies have gradually been developed to obtain the constitution of immune cells directly from tissue omics data (9, 10). Computational methods have several advantages over traditional experimental methods, including high throughput, automation, and the recognition of unknown cell types (11). Quantification of immune cells from omics data is an important strategy to infer the number and proportion of different immune cell subsets from high-throughput sequencing data. Previously, we have developed ImmuCC and seq-ImmuCC to infer the relative compositions of immune cell types in mouse tissues from microarray mRNA expression or bulk RNA-seq data (12, 13). These methods have been widely used to quantify the immune cell compositions of mouse tissues. However, these bulk expression profile-based methods for quantifying immune cells lack the ability to analyze individual cells and therefore cannot help effectively analyze immune cell heterogeneity.

Single-cell RNA sequencing (scRNA-seq) technology has emerged as a powerful technique for studying the heterogeneity and complexity of RNA transcripts within individual cells (14–16), and for identifying the composition of cell types and functions within different tissues, organs and organisms (17). The advancements in scRNA-Seq technology now allow us to obtain single cell gene expression and quantitative composition data of various immune cells in tissues. To identify immune cells in scRNA-Seq data, clustering followed by manual annotation is commonly used (18, 19). However, this strategy is usually labor-intensive and subjective with respect to the selection of signature genes, which causes poor reproducibility of cell annotation by different researchers. Moreover, some rare cell types cannot be directly clustered, and some abundant cell types with different anchor genes selected during integration can lead to undetermined annotation results.

In recent years, an increasing number of automated cell type annotation methods have been developed (20). These annotation methods can be categorized into three types: (1) marker gene-based methods, such as SCINA (21), scCATCH (22) and SCSA (23), which use prior knowledge for cell type annotation; (2) similarity-based methods, such as SingleR (24),which are based on correlations between query cells and predefined reference cell types, they assign the label of the type with maximum correlation; (3) supervised classification-based methods, scPred (25) using a combination of unbiased feature selection from a reduced-dimension space, and machine-learning probability-based prediction method to annotate cell types. Garnett (26) constructs a reference cell type hierarchy and uses elastic net regression for cell type prediction. Although these tools have provided powerful annotation performance for single cell sequencing data, their major limitation is the lack of specialized annotation tools for immune cells. Achieving consistent annotations for immune cell types is a challenge, as the same immune cell is often not in the same layer label in different tools. For instance, T cells belong to the major cell type, whereas CD4 T cells and CD8 T cells are subtypes of the former, the output of most tools is often a mixture of major cell types and subtypes. Moreover, the similar gene expression profiles of immune cell types cause difficulty in distinguishing them accurately. As a result, it is difficult to obtain accurate annotations and uniform output labels for immune cell types from scRNA-Seq data.

To address this issue, we have developed a hierarchical immune cell annotation method, namely sc-ImmuCC, based on the hierarchical lineage differentiation of immune cells. The core concept of sc-ImmuCC is to define the signature gene sets for differential immune cell types and to recognize them based on the enrichment scores of the signature genes. In sc-ImmuCC, the major types of immune cells are first annotated, followed by annotation of the subtypes of each cell type individually, which can reduce the interference between similar cell types, such as T cells and NK cells, and improve the accuracy of subtype annotation by avoiding cluttered annotation labels. The hierarchical annotation strategy of sc-ImmuCC can not only effectively distinguish immune cells with defined signature genes, but also provides an open framework for integrating more knowledge for future annotation.

## Materials and methods

### Summary of sc-ImmuCC

Briefly, sc-ImmuCC is a hierarchical method for annotating immune cell types based on signature gene sets and ssGSEA [the single-sample GSEA, an extension of Gene Set Enrichment Analysis (GSEA)]. Our objective is to collect representative and discriminative signature genes to the extent possible, and the genes are retained for later calculation. There are three main steps in the sc-ImmuCC model. The first step is signature gene selection. For the first layer of the immune cell types, canonical cell markers are mainly selected. For the second and third layers, not only the canonical cell markers, but also some functional feature genes that are highly expressed in RNA-Seq or scRNA-Seq data and some signature genes from the disease data sources in Ingenuity Pathway Analysis (IPA) are also included. The second step is calculation of the enrichment scores. According to the defined gene sets, the ssGSEA algorithm is used to calculate the immune cell enrichment scores of each cell hierarchically. Finally, according to the enrichment score, the cell types are annotated, and the largest score value is selected and converted into a cell type label.

### Hierarchical immune cell types

**Figure 1:**
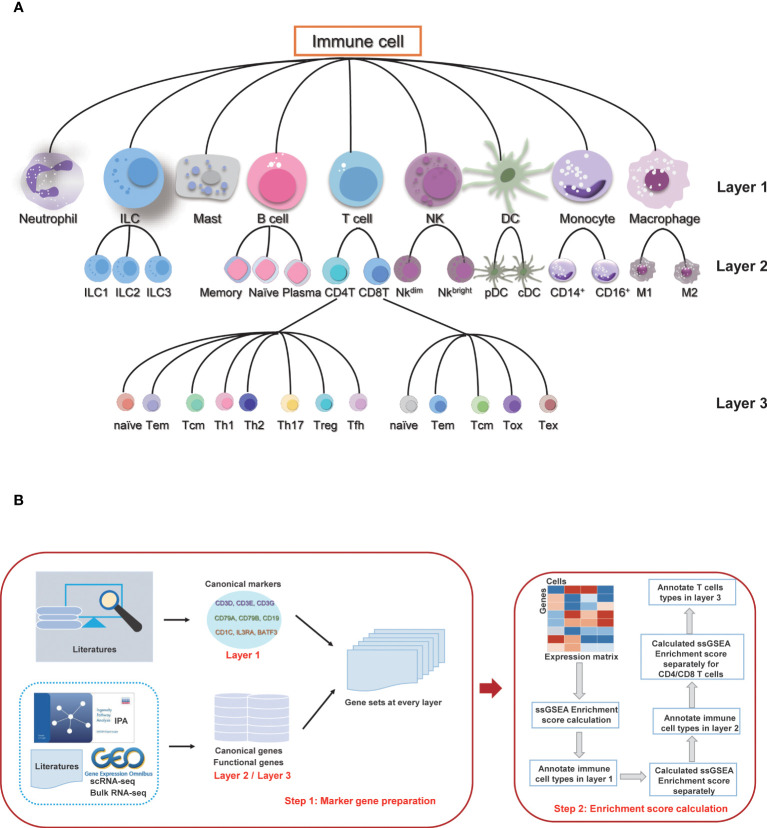
Overview of sc-ImmuCC. **(A)** Immune cell types and layers in sc-ImmuCC. **(B)** Signature gene sets for each cell type were collected and screened separately based on the cell types of different layers (left), and the process of sc-ImmuCC hierarchically annotating immune cells (right).

According to the differentiation lineage of the immune cells, we divided the annotation process of immune cells into three layers (**Figure 1A**). The first layer consists of nine major immune cell types: T cells, B cells, monocytes, macrophages, dendritic cells (DC), natural killer cells (NK), innate lymphoid cells (ILC), mast cells, and neutrophils. The second layer of cells is the subtype of the first layer of cells, mainly including the ILC subtypes: ILC1, ILC2 and ILC3; B cell subtypes: naïve B cells, memory B cells and plasma cells; T cell subtypes: CD4 T cells and CD8 T cells; NK subtypes: NK^_bright^ and NK^_dim^; DC subtypes: plasmacytoid DC (pDC) and conventional DC (cDC); monocyte subtypes: classic monocytes and non-classical monocytes; and macrophage subtypes: M1 macrophage and M2 macrophage. There are a total of 16 cell subtypes in the second layer. The third layer is a more specific subtype classification for the CD4 T cells and CD8 T cells in the second layer. The CD4 T cells include CD4_naive, CD4_central_memory, CD4_effector_memory, regulatory T cell (Treg), T follicular helper cell (Tfh), T helper (Th) cell 1, Th2 and Th17 subtypes, and the CD8 T cells include CD8_naive, CD8_central_memory, CD8_effector_memory, cytotoxic cells, and exhausted cells subtypes. The subsequent annotation process is performed in each layer separately.

### Definition of signature gene sets

The signature genes for each immune cell type were obtained by integrating the cell markers reported in the literature. Canonical experimentally validated signature genes were used as the marker genes for the first layer of cell types. Except to classical signature genes collected from the literature, signature genes derived from scRNA-seq, bulk RNA-seq data, and IPA were used as sources for the signature genes of the second and third layer cells (**Figure 1B**). Genes with low expression values were removed based on their average expression among multiple datasets. After filtering the low expressed marker genes, 11 gene sets were used to distinguish different immune cell types (**Tables S6-16**), including the signature gene sets used to distinguish immune and non-immune cells, immune cells of nine major types, namely B cells, T cells, macrophages, monocytes, neutrophils, mast cells, innate lymphoid cells, dendritic cells and natural killer cells. The details of signature gene sets (**Tables S6-16**) were shown in **Supplementary Data**.

### Dataset and preprocessing

All single cell gene expression datasets used in this study were obtained from public accessions, downloaded from the NCBI GEO, 10x Genomics, and EMBL-EBI ArrayExpress databases (**Table S1**). We used the original cell type annotation provided by each publication as ground truth, and systematically standardized the original labels. The specific original labels and their corresponding standardized labels can be found in **Table S3** (see **Supplementary Data**). For cells with ambiguous original annotations, such as “Mono/Mac”, “NK/T”, and “T/B doublets”, we discard them and retain only the cell type with a single annotation label.

**Figure 2:**
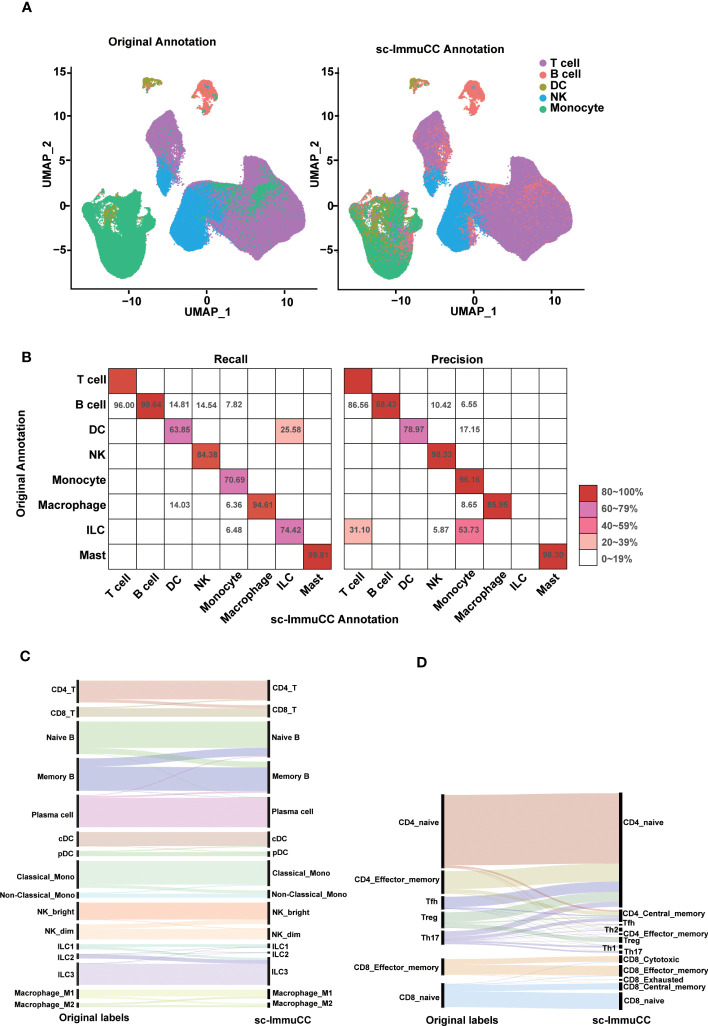
Evaluation of sc-ImmuCC on some annotated datasets. **(A)** UMAP plot the PBMC dataset with the original cell label (left) and the cell label annotated with sc-ImmuCC (right). **(B)** Pheatmap comparing the cell types from the original publication (rows) to those inferred by sc-ImmuCC (columns) for E-MTAB-11536. The color represents the recall and precision score (as a percentage) of each original cell type predicted by sc-ImmuCC. **(C)** Sankey plot for the sc-ImmuCC annotations on Layer 2. Cell type annotations by sc-ImmuCC (right) with the original cell type annotations in the dataset (left). **(D)** Sankey plot for sc-ImmuCC annotations on Layer 3.

To test the effectiveness and robustness of the method, 14 independent scRNA-Seq datasets were used, covering a wide range of immune cell types and tissue sources. For the PBMC dataset, the five common immune cell types, namely T cells, B cells, dendritic cells, monocytes, and natural killer cells, were retained, and some rare cell types such as platelets were removed from the testing dataset. Considering the imbalance in the E-MTAB-11536 dataset, we randomly sampled it for annotating the cell types in **Figure 2B**. For cell types with more than 50,000 cells, we randomly selected 12,000 cells for testing. For cell types with more than 20,000 and less than 50,000 cells, we randomly selected 8,000 cells. For cell types with less than 10,000 cells, we chose all of them to test (**Table S4**).

**Figure 3:**
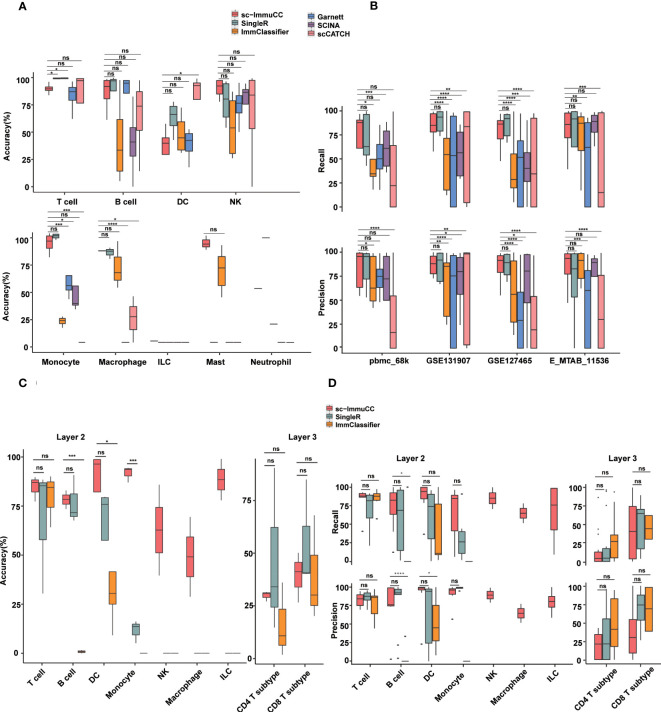
Performance of sc-ImmuCC in comparison with other methods. Each dataset was generated by randomly selecting 3000 cells per cell type. If the number of this type was less than 3000, we included all of them. Each dataset was randomly sampled five times; each tool was tested separately; and the results were averaged over five times. **(A)** Boxplots show the accuracy on layer 1 in different cell types. **(B)** Boxplots show the recall and precision score on layer 1 in different datasets. **(C)** Boxplots show the overall accuracy on layer 2 and layer 3 of sc-ImmuCC, SingleR and ImmClassifier. **(D)** Boxplots show the recall and precision on layer 2 and layer 3 of sc-ImmuCC, SingleR and ImmClassifier. t.test was conducted for sc-ImmuCC with other tools. *p < 0.05, **p < 0.01, ***p < 0.001, ****p < 0.0001. ns, not significant.

**Figure 4:**
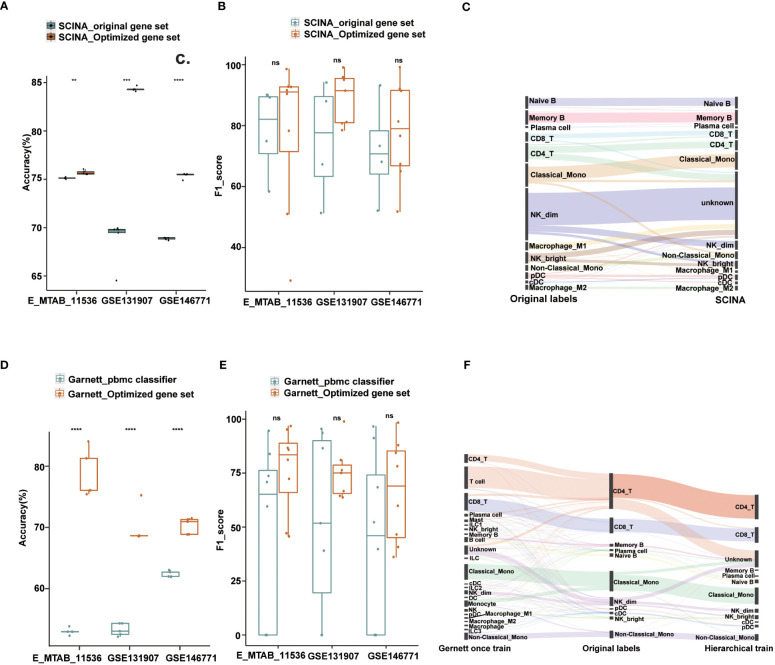
Performance of Garnett and SCINA with optimized gene sets and hierarchical annotations. **(A)** Overall accuracy comparison using SCINA’s original gene set (three cell types) and optimized gene set (nine cell types) in layer 1. **(B)** Boxplots show the F1-score of SCINA with different gene sets in layer 1. **(C)** Sankey plot of SCINA in layer 2. **(D)** Overall accuracy comparison using Garnett_pbmc classifier and Garnett train with optimized gene set in layer 1. **(E)** Boxplots show the F1-score of Gernett with different classifier in layer 1. **(F)** Sankey plot of Garnett using the hierarchical train and one-step train in layer 2. t.test was conducted for Optimized gene set with original gene sets. **p < 0.01, ***p < 0.001, ****p < 0.0001. ns, not significant.

All the data used in the tests without any filtering, correction, or normalization. For the datasets used in **Figures 3** and **4**, non-immune cells were removed in advance if they were included, without any additional processing. In the testing **Figures 3** and **4**, the dataset GSE131907 underwent pre-filtering to exclude non-immune cells, and the original data was directly used for testing in **Figure S5B**.

### Reference cell type annotation tools

We selected five existing methods for performance comparison with sc-ImmuCC: SingleR, ImmClassifier, Garnett, SCINA and scCATCH. SingleR is a similarity-based method; ImmClassifier and Garnett are based on machine learning methods, and are both based on hierarchy. SCINA and scCATCH are marker gene-based methods. The former is based on a custom gene set, and the latter is based on the marker gene database.

To perform the tool comparison, ImmClassifier was run with default parameters. We ran SingleR with default parameters and ran Garnett with the hsPBMC pretrained classifier. The SCINA R package using precompiled immune cell signatures from the RCC patients, and we labeled cell types which not included in the precompiled gene set as “unknown”. scCATCH was used with default parameters, and we choose the “Bone”, “Lung” and “Blood” tissue sources.

To balance the datasets and randomness of the methods, we adopted a random sampling method for each of the four datasets. We randomly selected 3,000 cells per cell type (>3,000 cell) each time; if there were less than 3,000 cells per cell type, all of them were included each time (**Table S5**). Five random samplings were carried out in total, and the final results were the average of the five.

### Statistical analysis and visualization

**Figure 5:**
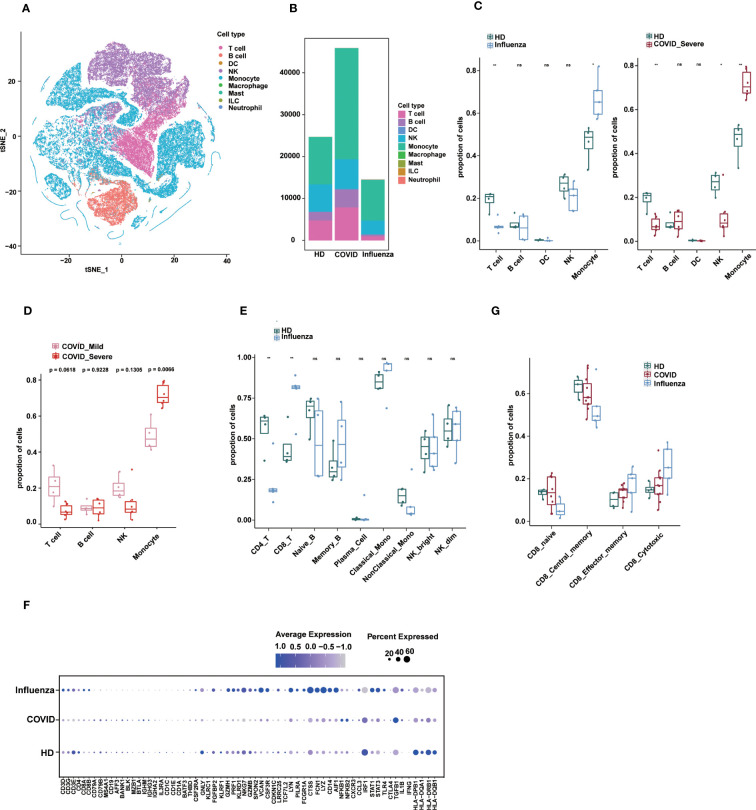
Applications of sc-ImmuCC. **(A)** tSNE plot colored by annotated cell types. **(B)** Stacked diagram of cell composition for each group. **(C)** Boxplots show the cell proportion of each group in layer 1. T tests were conducted for each cell type between the disease and HD groups. *P < 0.05, **P < 0.01, and ***P < 0.001. **(D)** Fraction of cell types in COVID_Mild and COVID_Server in layer 1. **(E)** Cell proportion between the influenza and HD groups in layer 2. **(F)** The dot plot showing the marker expression profiles of different groups under sc-ImmuCC annotation. **(G)** CD8 T cell subtypes proportion between the disease and HD groups.

The basic statistical analyses presented in **Figures 4** and **5** were performed with the “ggpubr” package. UMAP plots were obtained by “Seurat” package (27, 28). For the datasets GSE144744 and GSE131907, the top 2,000 most variable genes were used for PCA. The top 15 principal components were used to generate the UMAP and tSNE plots. Sankey plots were generated using “networkD3” package (29). Boxplots were generated using “ggplot2” package (30). Pheatmaps are drawn with the “pheatmap” package.

The Seurat R package is integrated into sc-ImmuCC as a visualization tool to help users gain a more intuitive understanding of the annotation results and gene expression in the data. Seurat was not used as a data preprocessing tool. No clustering was performed using Seurat. Seurat was employed for principal component analysis of the data and added the annotated cell types to the Seurat object. The annotation results were visualized in UMAP and tSNE plots based on PCA and annotated cell types.

### Input and output of sc-ImmuCC

The input for sc-ImmuCC is a single-cell count matrix with cells unique barcodes as column names and gene names as row names. The input matrix does not require any filtering, correction, or normalization. However, users have the option to perform these operations if desired. From our testing, we found that normalization does not affect the annotation results.

The output of sc-ImmuCC is a CSV file containing the annotation results for each cell type at the first, second, and third layer, along with corresponding tSNE, UMAP, DotPlot, and Heatmap plots. For second and third-layer annotations, if the cell number for a specific cell type is less than 50, graphical representation is omitted, and only the CSV annotation file is provided.

### Codes and availability

Codes and scripts of the sc-ImmuCC method were written in R version 4.1.1 and Bioconductor (31) version 3.14, installation instruction, usage and example codes can be found at https://github.com/wuaipinglab/scImmuCC. The ssGSEA enrichment scores were calculated with the “GSVA” package (31). The relevant images are drawn by the “Seurat” package.

## Results

### Overview of the sc-ImmuCC method

The stepwise differentiation of immune cells in nature provides a good reference framework to identify immune cell types from single-cell sequencing data hierarchically. By simulating as the hierarchical differentiation of immune cells, here we propose sc-ImmuCC, a tool to annotate the immune cell types within scRNA-seq data. The immune cell types included in sc-ImmuCC can be hierarchically classified into three layers according to the differentiation lineage trees, with nine major cell types in the first layer, 16 cell subtypes in the second layer, and 13 T cell subtypes in the third layer (**Figure 1A**).

Cell differentiation is primarily driven by differential gene expression, leading to the formation of a diverse range of cell types. To identify specific cell types, it is crucial to identify the signature genes that are highly expressed in each cell type. sc-ImmuCC aims to identify immune cell types in scRNA-Seq data by screening the signature gene sets of different cell types, calculating the enrichment scores hierarchically through the ssGSEA algorithm, and providing annotations based on the scores. Three key steps are included in the sc-ImmuCC model (**Figure 1B**, see Methods for more details. First, we conducted expression screening of all gene sets and select genes with higher average expression across multiple datasets as signature gene sets (**Figures S1B, C**). Second, we hierarchically identified nine major immune cell types and their subtypes (**Figure 1A**). Third, to evaluate the robustness and applicability of the method, we applied it to different datasets, including PBMC datasets, enriched immune cell datasets, and tumor tissue datasets.

### Performance of sc-ImmuCC on immune cell datasets

The performance of sc-ImmuCC in the first layer was evaluated on two different datasets. On a testing PBMC dataset, sc-ImmuCC achieved an overall accuracy of 85%, and the accuracy of T cells, B cells, DCs, NKs and monocytes were 86%, 99%, 75%, 92%, and 76%, respectively (**Figure 2A**). Some labelled NK cells were annotated as T cells in our model for their highly expressed CD3D, CD3E and GNLY (**Figure S2A**), which suggests that our method may can correct some mis-annotations. Similarly, in the T cell-dominated cluster, a small fraction of monocytes was relabeled as B cells by sc-ImmuCC due to their highly expressed CD79A and CD79B but not LYZ (**Figure S2A**).

To further assess the performance of sc-ImmuCC in annotating complex datasets, an immune-enriched dataset consisting of 16 tissues was selected. We compared the annotation results of sc-ImmunCC with the manually curated annotations of the original paper. In the first layer, sc-ImmuCC achieved an overall accuracy of 88% (**Figure 2B**). The F1-score of T cells, B cells, DCs, NK cells, monocytes, macrophages and mast were 91%, 94%, 71%, 91%, 81%, 90%, and 99%. However, there were relatively high rates of misclassification in the innate lymphoid cells, with 31% of those cells labelled as T cells and 53.7% as monocytes (**Figure 2B**). Upon examining the expression levels of some known marker genes associated with innate lymphoid cells in this cell cluster, it was observed that the expression of these genes was very low in most cells (**Figure S2B**). Hence, we performed a secondary evaluation of sc-ImmuCC’s performance in annotating ILC cells using the GSE146771 dataset. The results demonstrated an annotation accuracy of over 50% for ILC cells in this dataset (**Figure S2C**). The distinction between the GSE146771 dataset and the dataset depicted in **Figure 2B** lies in the higher expression levels of signature genes associated with ILC cells in the former (**Figure S2D**). This suggests that sc-ImmuCC did not accurately identify the innate lymphoid cells in E_MTAB_11536 dataset due to their low expression of the signature genes.

At the second layer, the sc-ImmuCC demonstrates a high level of annotation accuracy for T cells, B cells, DCs, NKs, monocytes, macrophages and ILCs subtypes (**Figure 2C**), with an overall accuracy rate of 78-90%, except for macrophages which had an accuracy rate of 70% (**Figure S2C**). As expected, the performance of sc-ImmuCC decreased at the third layer for the CD4 and CD8 T cells. Though most subtypes of the CD8 T cells could be accurately annotated, the overall accuracy for the CD4 T cell subtypes decreased significantly (**Figure 2D**). This may suggest that further optimization of gene sets for CD4 T cell and CD8 T cell subtypes to improve discrimination is needed, as many signature genes do not exist in the scRNA-Seq data. Besides, the original literature annotation for subtypes may not be accurate, as the signature gene expression of CD4 T cell subtypes is not clearly evident based on the original annotations. For example, IL17A, IL17F, RORA, and RORC were not significantly expressed in Th17 (**Figure S2D**).

### Comparison of sc-ImmuCC with other methods

To further assess the performance of sc-ImmuCC, we compared it with five representative methods for single cell annotation: ImmClassifier (32), Garnett, SingleR, SCINA, and scCATCH. The reference methods were chosen as the representative ones from hierarchical-based, correlation-based, and gene-based approaches. All methods were used on four independent scRNA-Seq datasets covering various tissue sources, including PBMC, metastatic lung adenocarcinoma, and non-small-cell lung cancer. All methods were compared using four metrics, namely overall accuracy, precision, recall, and F1-score.

Overall, almost all methods performed well at the first layer, sc-ImmuCC achieved an average overall annotation correctness of 71%, 80%, 85%, and 79% on the four datasets, respectively. It is worth noting that, although our tool achieved only 71% accuracy on the pbmc_68k dataset, all tools generated annotations for this dataset that deviated from the original ones. The best-performing method on this dataset, SingleR, achieved 73% accuracy (**Figure S3A**). Apart from a slightly lower overall accuracy observed for DC cells, the accuracy of sc-ImmuCC for other cell types exceeded 85% (**Figure 3A**). sc-ImmuCC demonstrated achieves comparable performance to other tools on the four datasets (**Figure 3B**). Except for the pbmc_68k dataset, the F1-score on tumor datasets and cross-tissue datasets outperformed other tools (**Figure S3B**).

At the second and third layers, we compared sc-ImmuCC with the ImmClassifier and SingleR methods. sc-ImmuCC demonstrated comparable accuracy for T cells, B cells and DC cells as SingleR and ImmClassifier (**Figures 3A, B**). sc-ImmuCC exhibited significantly superior annotation performance for some other cell types, such as dendritic cells, monocytes, and macrophages (**Figures 3C, D** and **S3C, D**). Due to the hierarchical annotation strategy, sc-ImmuCC reduced the interference of some similar signature gene expressions between cell types from different branches. At the third layer, sc-ImmuCC outperformed the other two methods in identifying the subtypes of CD8 T cells, but its performance for the CD4 T cells was equally limited as of ImmClassifier and SingleR (**Figures 3C, D** and **S3E**).

### Optimized gene sets and hierarchical annotation facilitate other tools

To assess the impact of gene set optimization and hierarchical annotation on other tools, we applied these two strategies to two methods, SCINA and Garnett. SCINA is a marker-based annotation method that uses precompiled immune cell signatures from RCC patients, including classical monocytes, CD19_B and NK_dim cell types, and other cell types not included may be annotated as “unknown”. With our defined first layer signature gene sets, which included nine cell types, SCINA achieved a significant improvement in the overall annotation accuracy. For the lung adenocarcinoma and colorectal cancer datasets, the accuracy improved by 6% and 15%, respectively, and our method exhibited a comparable integrated performance to that of the original gene set in the cross-tissue datasets (**Figures 4A, B**). Subsequently, we used the second layer signature gene set on SCINA and found that almost all subtypes could be identified except for the M1 macrophage and natural killer cell subtypes (**Figure 4C**). Particularly, the precision for the subtypes of B cells and monocytes exceeded 75%. The recall for the B cell subtypes is also exceeded 91% (**Figure S4A**).

Similar results were also observed when applying the optimized gene set and hierarchical annotation to Garnett. By using our first layer gene sets with Garnett, the accuracy improved by 8%-26% (**Figure 4D**). Not only did the number of annotation types increase, but also the overall performance has also improved (**Figure 4E**). For the second layer annotation, we first integrated the optimized gene sets to create a comprehensive gene set for one-step training. Additionally, we trained classifier with each gene set separately to achieve a hierarchical annotation. By comparing the annotated results with those from the original literature, we found that the hierarchically trained classifier had better annotations for most cell subtypes. In contrast, the one-step-trained classifier assigned most cells as first layer immune cell types and failed to obtain finer annotations (**Figure 4F**). Except for NK^_bright^, the recall for most subtypes exceeded 90%, and the precision rate for the DC and monocyte subtypes was more than is over 80% (**Figures S4B, C**).

These results demonstrate that the strategies of optimized gene sets and hierarchical annotation can not only identify more immune cell types, but also generate more accurate annotation results. Overall, our results highlight the potential of these strategies for improving the accuracy and completeness of cell type annotation in scRNA-seq data, even when the strategies are applied to other annotation methods.

### Immune-cell profiling in COVID-19 and influenza using sc-ImmuCC

We employed sc-ImmuCC to annotate COVID-19 data (33) to gain insights into cellular composition and immune responses across various pathologies. A previous study examined the single-cell transcriptome of PBMCs from COVID-19 and influenza patients and presented a comprehensive overview of their immunophenotypes at the single-cell level (34). However, by focusing on some subset of immune cell types, the study did not fully labelled immune cell subsets. To address this, we re-annotated this dataset and conducted an in-depth analysis of immune cell composition across different pathological states.

We identified five major cell types, namely T cells, B cells, dendritic cells, monocytes, and NK cells, along with their subtypes (**Figures 5A** and **S5A**). Due to the small number of DC cells, we only annotated them at the first layer. The relative proportions of immune cells in PBMCs from the disease groups were altered compared to those in healthy donors (**Figures 5B, C**). Interestingly, severe COVID-19 and influenza showed some similarity in terms of a significant increase in monocyte proportion compared to healthy donors (**Figures 5C** and **S5B**), which is consistent with the literature, with subtle differences in DCs. In severe COVID-19, the proportion of monocytes was significantly increased, whereas the proportions of T cells and NK cells were decreased (**Figure 5C**). The proportion of monocytes was also significantly higher in severe COVID-19 patients compared to mild COVID-19 patients (**Figure 5D**). In influenza, the proportion of CD8 T cells was significantly increased, and CD4 T cells were significantly decreased (**Figure 5E**).

The expression profiles of immune cell markers in COVID-19, influenza patients, and healthy donors showed that common monocyte markers such as LYZ, VCAN and FCN1 were more significantly expressed in influenza patients, which may reflect stronger pro-inflammatory signals in influenza patients. Some inflammation-related genes such as *NFKB1, NFKB2*, and *TGFB1* were specifically upregulated in COVID-19, whereas genes such as *STAT3, STAT1, TLR4, NLRP3*, and *PYCARD* were specifically upregulated in influenza (**Figure 5F**). In addition, our results provide annotation of NK cell subtypes that were not previously identified in the literature.

Finally, we observed differences in the composition of CD8 T cell subtypes between healthy and disease states (**Figure S5C**). In the GSE149689 dataset, the proportion of CD8 central memory cells was slightly lower in the COVID-19 and influenza groups compared to the healthy group, whereas CD8 cytotoxic cells showed the opposite trend, although not significant (**Figure 5G**). Nevertheless, sc-ImmuCC allowed for the annotation of subtypes at every hierarchical layer and was effective in annotating diverse source datasets. Applying it to a tumor dataset demonstrated its ability to accurately distinguish between immune cells and non-immune cells (**Figure S5D**).

## Discussion

Owing to the bias and uncertainty of the scRNA-Seq technology in detecting the biological characteristics of immune cells, accurately identifying immune cells and improving the accuracy of recognition is critically important. In this study, we designed a method called sc-ImmuCC to identify immune cell types from scRNA-Seq data. The performance of sc-ImmuCC was validated on various independent datasets with good robustness and accuracy. Compared with similar existing tools, sc-ImmuCC provides a hierarchical annotation for immune cells, which has several benefits. First, the concept of hierarchy is based on simulating the natural differentiation of immune cells. In the human body, hematopoietic stem cells (HSCs) continuously replenish all types of blood cells through a series of lineage-restricted steps. The immune cells are classified by lineage stratification, which allows for a more comprehensive annotation of the corresponding cell types. Second, hierarchical annotation avoids interference from similar gene expression profiles by not comparing them between different branches. Finally, hierarchical annotation is an open computational framework that can be continuously integrated and extended to new immune cell types in the future.

Moreover, optimized gene sets and hierarchical strategies can also improve the performance of other similar methods. For example, although Garnett is hierarchical, its trained classifiers do not provide proper output labels for some cell types, which makes it difficult for users to obtain a simple and clear result. Using the hsPBMC pretrained classifier will output T cells, CD4 T cells, and CD8 T cells, which are not at the same layer, whereas using the hsLung pretrained classifier cannot distinguish dendritic cells, monocytes, and macrophages. For users who want to understand the specific composition of these cells in the data in detail, this may be inconvenient. However, after using the optimized gene set to self-train with Garnett, we can not only distinguish dendritic cells, monocytes, and macrophages well in the first layer, but also train each subtype of cells separately and then perform hierarchical annotation with the dataset, thus greatly improving the annotation performance of each subtype of cells. Hierarchical annotation reduces errors in identifying cell subtypes, whereas gene set selection simplifies complex gene data to highlight the relevant genes. However, gene set selection may miss some relevant genes due to differences in regulation and tissue specificity. To improve accuracy, multiple tools should be used in hierarchical annotation and diverse strategies should be applied in gene set selection, such as utilizing diverse gene expression datasets, biological pathway databases, and functional enrichment analysis tools to cover a wide range of signature genes comprehensively.

Using the sc-ImmuCC method, we can effectively distinguish immune cells and non-immune cells in healthy and disease data, and quantify the composition of major immune cells across different tissues or organs in human scRNA-Seq data. An example is to use sc-ImmuCC to annotate immune cells in COVID-19 patients. With a large number of clinical and laboratory studies published on COVID-19 (35, 36), “omics” approaches have played an important role in the study of COVID-19 and generated massive amounts of data at an unprecedented rate (37). Accurately identifying immune cell types in COVID-19 patients and dissecting the immune response in COVID-19 may aid the development of vaccines and antiviral drugs (38). sc-ImmuCC can accurately identify immune cell types hierarchically, thus providing a more precise understanding of cell subtypes and enabling more accurate comparisons of the changes in the cell composition and gene expression of interest across different diseases. By identifying immune cells in different pathological states, valuable immune cell background information can be extracted, thus providing more reliable evidence for the diagnosis and treatment of diseases.

Although using sc-ImmuCC to annotate the subtypes of cells at the third layer is not yet perfect, we hope that this study can promote the development of algorithms that can achieve this goal. In the future, we will continue to improve the annotation for the CD4 and CD8 T cell subtypes, such as optimizing gene sets, assigning weights to genes in the corresponding cell type based on their contribution values in the gene expression matrix, and integrating other methods. For the selection of gene sets, we will consider selecting genes according to the source of the data, and testing whether there are tissue-specific differences in the gene sets. Another limitation of sc-ImmuCC is that cannot detect new cell types. It is an annotation method based on a given specific cell type gene set and assigns cells to the cell type with the highest enrichment score calculated. This limitation needs to be addressed in future work, and for unidentifiable types, they can be improved by being identified as “unknown”. Currently, our method cannot fully annotate all immune cell types, such as γδ T cells, which are known to play a crucial role in tumor defense, were not included in our signature gene set. In addition, some cell types with a small proportion, such as eosinophils and basophils, were also not included due to too few datasets to test. Consequently, sc-ImmuCC has limitations when studying these less common immune cell types due to the absence of appropriate test datasets for certain uncommon cell types. In the context of certain allergic diseases or parasitic infection sequencing data, the cell types annotated by our method may not possess sufficient accuracy and comprehensiveness for practical utilization. Therefore, further refinement of our method by including more cell types in the signature gene sets and finding more available test datasets will be an important future direction. Most of the previous research including our own method, can only identify differentiated terminal cells but cannot annotate immune cells undergoing continuous differentiation. In the future, it may be possible to construct a reference tree of immune cell evolution and project cells directly onto the tree to obtain their specific location in the differentiation path, to better understand the distribution and function of immune cells for a comprehensive and accurate study of immune cells.

Overall, the performance of sc-ImmuCC mainly depends on the given signature gene sets currently. The public datasets of immune cells were collected from different sources and tissues. If we further divide each subtype according to different tissue sources, perhaps there can be more accurate annotation of immune cells in scRNA-Seq data. The study of precise identification of immune cell types holds scientific significance and clinical application prospects, and further research will promote immunology’s development.

## Data availability statement

The original contributions presented in the study are included in the article/**Supplementary Material**. Further inquiries can be directed to the corresponding author.

## Author contributions

YJ, ZC and AW conceived and designed the study. YJ collected the data and preprocessed it. YJ, ZC and AW analyzed the data and results. NH and JS contributed to the discussion and analysis of the studies. YJ, ZC and AW wrote the paper. All authors contributed to the article and approved the submitted version.
