# Supplementary material for: sc-ImmuCC: hierarchical annotation for immune cell types in single-cell RNA-seq
Source: Front Immunol. 2023 Jul 20;14:1223471. doi: 10.3389/fimmu.2023.1223471 (PMC10399579; doi:10.3389/fimmu.2023.1223471)
Supplement: Supplementary file 1 [file DataSheet_1.docx]

Supplementary Material

sc-ImmuCC: Hierarchical annotation for immune cell types in single-cell RNA-seq

**Ying Jiang^1,2†^, Ziyi Chen ^1,2†^, Na Han^1,2^, Jingzhe Shang^1,2^, Aiping Wu^1,2*^**

First Author*, Co-Author, Co-Author

*** Correspondence:** Corresponding Author: Aiping Wu

E-mail: wap@ism.cams.cn

# Supplementary Figures and Tables

**.** **Supplemental Tables**

**Table S1: GEO Accessions for Published Datasets Included in Manuscript**

| Accession | Repository | Reference |
| --- | --- | --- |
| E_MTAB_11536 | ArrayExpress | Conde et al. 2022 |
| GSE131907 | Gene Expression Omnibus | Kim et al. 2020 |
| GSE127465 | Gene Expression Omnibus | Zilionis et al. 2019 |
| GSE130157 | Gene Expression Omnibus | Griffiths et al. 2020 |
| GSE144744 | Gene Expression Omnibus | Kaufmann et al. 2021 |
| GSE146771 | Gene Expression Omnibus | Zhang et al. 2020 |
| GSE161801 | Gene Expression Omnibus | Tirier et al. 2021 |
| GSE145926 | Gene Expression Omnibus | Liao et al. 2020 |
| GSE150050 | Gene Expression Omnibus | Mazzurana et al. 2021 |
| GSE136831 | Gene Expression Omnibus | Adams et al. 2020 |
| GSE115469 | Gene Expression Omnibus | MacParland et al. 2018 |
| GSE154763 | Gene Expression Omnibus | Cheng et al. 2021 |
| PBMC_68K | 10x Genomics Datasets | Zheng et al. 2017 |
| GSE149689 | Gene Expression Omnibus | Lee JS et al. 2020 |

**Table S2: Full name of cell types in cell type hierarchy.**

| Abbreviation | Full name |
| --- | --- |
| DC | dendritic cell |
| NK | natural killer cell |
| ILC | innate lymphoid cell |
| Mast | Mast cell |
| pDC | plasmacytoid dendritic cell |
| cDC | conventional dendritic cell |
| Mono CD14^+^ (classical_Mono) | CD14^+^ Monocyte (classical Monocyte) |
| Mono CD16^+^ (non-classical_Mono) | CD16^+^ Monocyte (Non-classical Monocyte) |
| CD4 T | CD4 T cell |
| CD8 T | CD8 T cell |
| CD4 T naïve | CD4 naïve T cell |
| CD4 Tem | CD4 effector memory T cell |
| CD4 Tcm | CD4 central memory T cell |
| CD4 Th1 | T helper cell 1 |
| CD4 Th2 | T helper cell 2 |
| CD4 Th17 | T helper cell 17 |
| Treg | regulatory T cell |
| Tfh | T follicular helper cells |
| CD8 naïve | CD8 naïve T cell |
| CD8 Tem | CD8 effector memory T cell |
| CD8 Tcm | CD8 central memory T cell |
| CD8 Tox | CD8 cytotoxic T cell |
| CD8 Tex | CD8 exhausted T cell |

**Table S3:** **Correction of cell labels.**

| Original Labels | Revise Labels | | |
| --- | --- | --- | --- |
|  | Layer 1 | Layer 2 | Layer 3 |
| T | T cell | / | / |
| T_Cell_CD4_CM | T cell | CD4_T | CD4_Central_memory |
| T_Cell_CD4_EM | T cell | CD4_T | CD4_Effector_memory |
| T_Cell_CD4_Naive | T cell | CD4_T | CD4_Naive |
| T_Cell_CD4_Tfh | T cell | CD4_T | Tfh |
| T_Cell_CD4_Treg | T cell | CD4_T | Treg |
| T_CD4_naive | T cell | CD4_T | CD4_Naive |
| CD4+ T Helper2 | T cell | CD4_T | Th2 |
| CD4+/CD45RA+/CD24-Naive T | T cell | CD4_T | CD4_Naive |
| CD4+/CD45RO+ Memory | T cell | CD4_T | / |
| CD8+ Cytotoxic T | T cell | CD8_T | CD8_Cytotoxic |
| CD8+/CD45RA+ Naïve Cytotoxic | T cell | CD8_T | / |
| Naïve CD4+ T | T cell | CD4_T | CD4_Naive |
| Tfh | T cell | CD4_T | Tfh |
| Th17 | T cell | CD4_T | Th17 |
| Tregs | T cell | CD4_T | Treg |
| T cells regulatory | T cell | CD4_T | Treg |
| T cells follicular helper | T cell | CD4_T | Tfh |
| CD8_ex | T cell | CD8_T | CD8_Exhausted |
| T_Cell_CD8_CM | T cell | CD8_T | CD8_Central_memory |
| T_Cell_CD8_EM | T cell | CD8_T | CD8_Effector_memory |
| T_Cell_CD8_Naive | T cell | CD8_T | CD8_Naive |
| T_CD8_naive | T cell | CD8_T | CD8_Naive |
| T_CD8_tox | T cell | CD8_T | CD8_Cytotoxic |
| Naïve CD8+ T | T cell | CD8_T | CD8_Naive |
| Cytotoxic CD8+ T | T cell | CD8_T | CD8_ Cytotoxic |
| Exhausted CD8+ T | T cell | CD8_T | CD8_ Exhausted |
| B_im | B cell | / | / |
| B | B cell | / | / |
| CD19+ B | B cell | / | / |
| Mem B | B cell | Memory_B | / |
| Naïve B | B cell | Naïve_B | / |
| Plasma cells | B cell | Plasma_Cell |  |
| B_Cell_Memory | B cell | Memory_B |  |
| B_Cell_Naive | B cell | Naive_B |  |
| Dendritic_Cell | DC | / | / |
| Plasmactiod_Dendritic_Cell | DC | pDC | / |
| CD141 + DCs | DC | cDC | / |
| CD1c + DCs | DC | cDC | / |
| pDCs | DC | pDC | / |
| hM02_pDC_LILRA4 | DC | pDC | / |
| hM03_cDC2-CD1C | DC | cDC | / |
| hM04_cDC1-batf3 | DC | cDC | / |
| Dendritic | DC | / | / |
| cDC1 | DC | cDC | / |
| cDC2 | DC | cDC | / |
| pDC_LILRA4 | DC | pDC | / |
| cDC1_CLEC9A | DC | cDC | / |
| cDC2_CD1C | DC | cDC | / |
| CD56+ NK | NK | / | / |
| NK_Cell_Active | NK | / | / |
| NK_Cell_Resting | NK | / | / |
| NK_dim | NK | NK_dim | / |
| NK_bright | NK | NK_bright | / |
| NK_CD16+ | NK | NK_dim | / |
| NK_CD56bright_CD16- | NK | NK_bright | / |
| Mono-CD14 | Monocyte | Classical_Mono | / |
| Mono-CD16 | Monocyte | NonClassical_Mono | / |
| CD14+  Monocyte | Monocyte | Classical_Mono | / |
| Monocyte_ Classical | Monocyte | Classical_Mono | / |
| Monocyte_Non-Classical | Monocyte | NonClassical_Mono | / |
| Inflammatory_Macropahge | Macrophage | Macrophage_M1 | / |
| Non-Inflammatory_Macropahge | Macrophage | Macrophage_M2 | / |
| Macropahges M1 | Macrophage | Macrophage_M1 | / |
| Macropahges M2 | Macrophage | Macrophage_M2 | / |
| ILC1 | ILC | ILC1 | / |
| ILC2 | ILC | ILC2 | / |
| ILC3 | ILC | ILC3 | / |
| Mast | Mast | / | / |
| MAST | Mast | / | / |
| Mast cells activated | Mast | / | / |
| Mast cells resting | Mast | / | / |
| Neutrophils | Neutrophil | / | / |

**Table S4: The number of cells sampled to test for E_MTAB_11536 in Figure2**.

| Cell number (N) | Sampled cell number per cell type |
| --- | --- |
| N < 5000 | all |
| 20000 < N < 50000 | 8000 |
| N > 50000 | 12000 |

**Table S5: The number of cells sampled to test for tools Comparison in Figure3.**

| Cell number (N) | Sampled cell number per cell type |
| --- | --- |
| N < 3000 | all |
| N > 3000 | 3000 |

**Supplemental Figures**

**
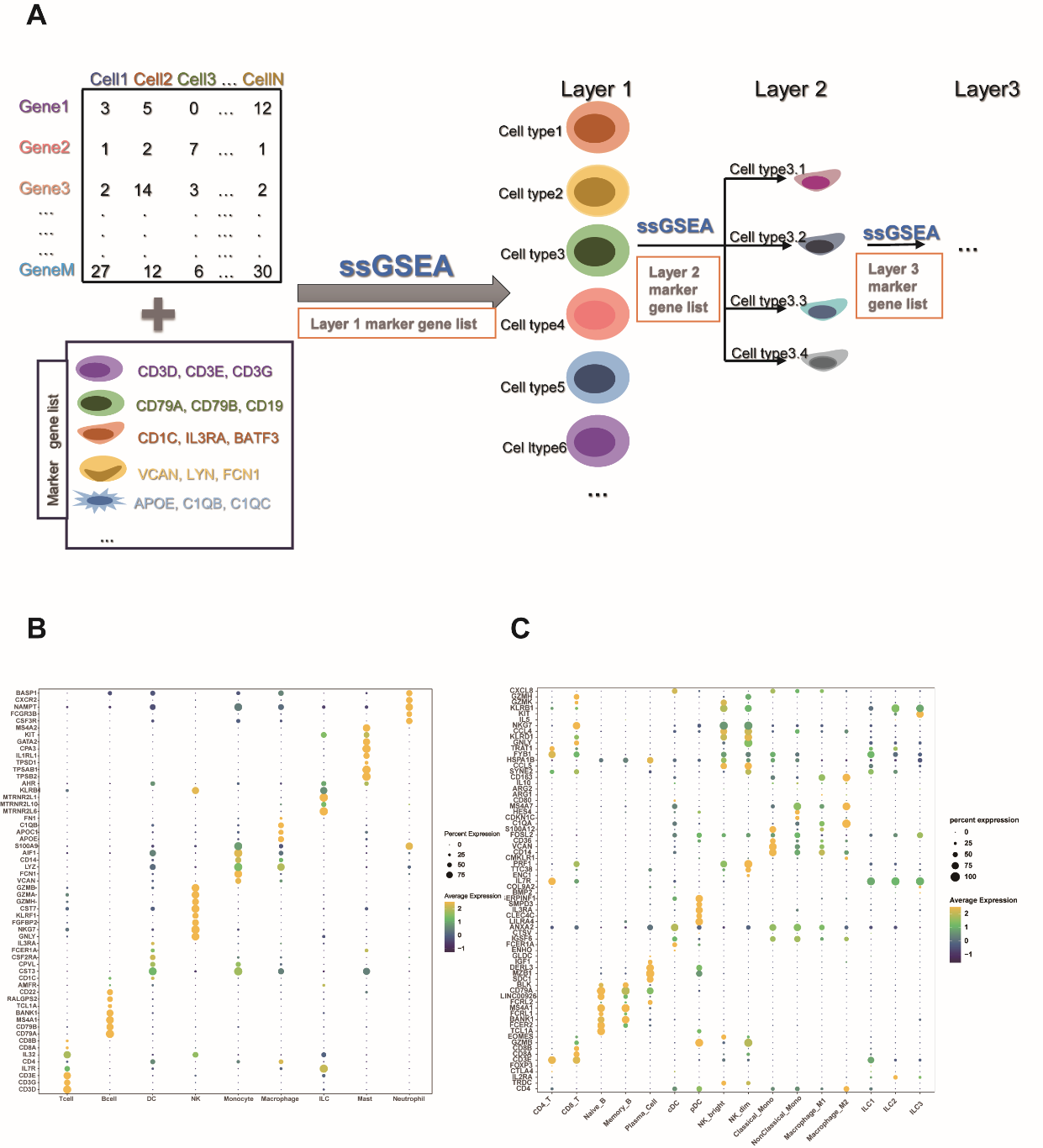
**

**Supplemental Figure 1.** Summary of sc-ImmuCC. (A) Workflow of sc-ImmuCC. (B), and (C) are heatmaps of expression of first layer and second layer signatures genes in multiple datasets.

**
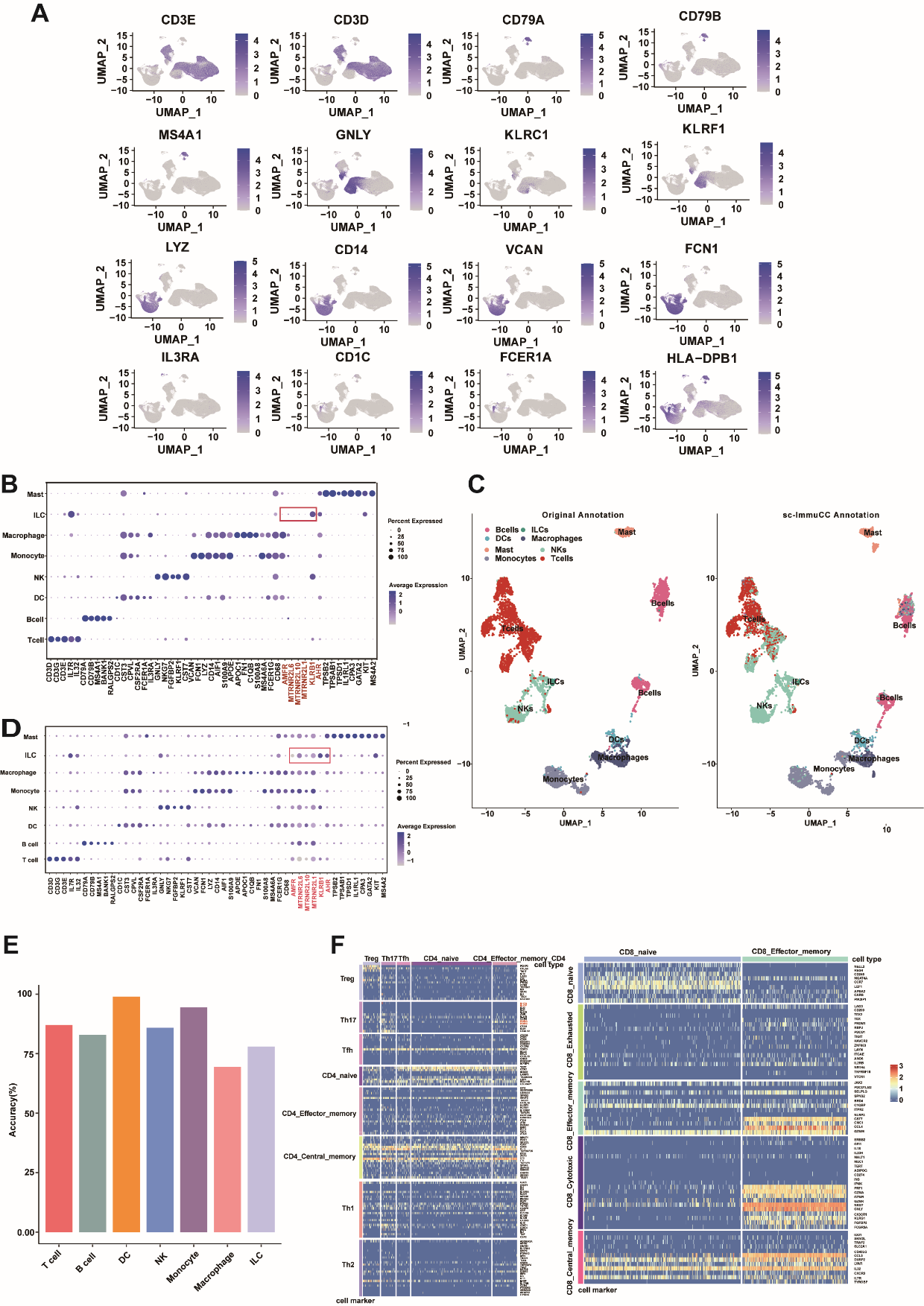
**

**Supplemental Figure 2.** Performance of sc-ImmuCC on real datasets. (A) Feature plot of PBMC dataset. (B) Gene expression of e-mtab-11536 dataset labelled by original. (C) Performance of sc-ImmuCC on the second layer. (D) The gene expression heatmap annotated by the original data, the row is the marker type, and the column is the cell type. The left are CDT T cells and the right are CD8 T cells.


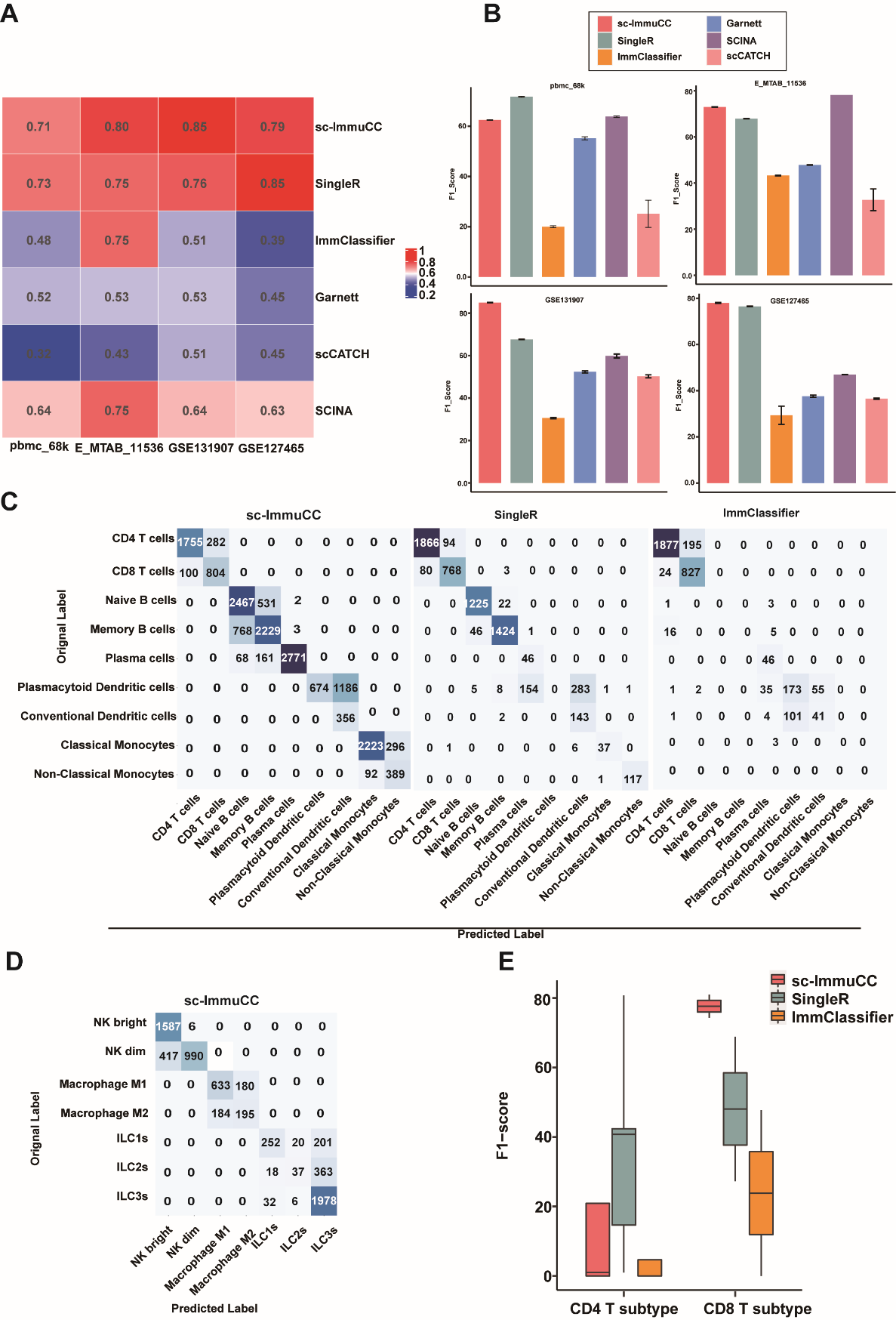


**Supplemental Figure 3.** Comparison of scRNA-Seq annotation methods. (A) Heatmap of the overall accuracy of the six methods at layer 1. (B) The average F1-score of the six methods in the four datasets tests at layer 1. (C) Confusion matrix showing annotation results for common subtypes at layer 2 by the sc-ImmuCC, SingleR and ImmClassiifer, with rows representing original labels and columns representing annotated labels. (D) Confusion matrix showing annotation results for unique subtypes at layer 2 to sc-ImmuCC. (E) The average F1-score of the three methods at layer 3.


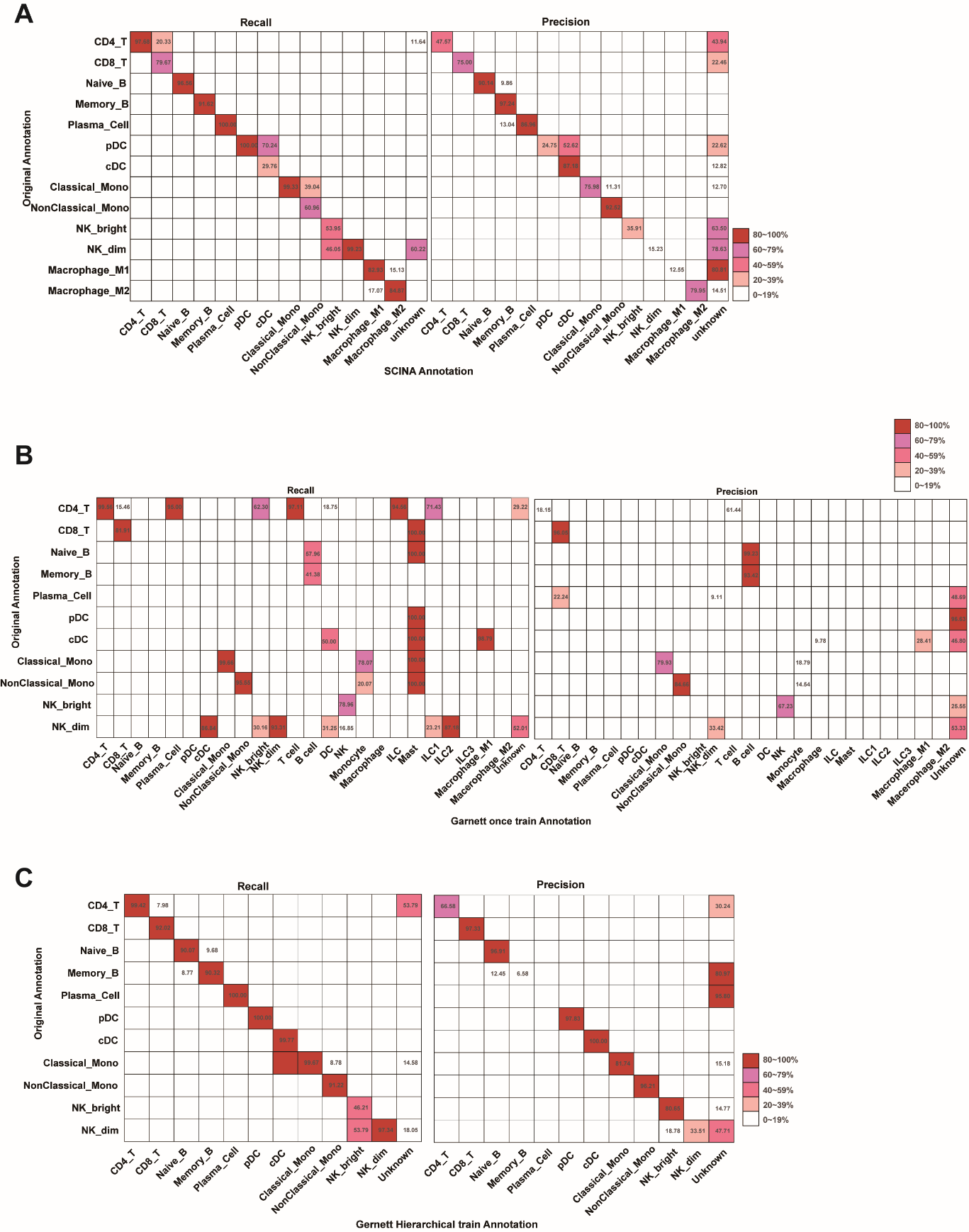


**Supplemental Figure 4.** Applying hierarchical annotation and optimization of gene sets to Garnett and SCINA. These heatmaps compare the cell types from the original publication (rows) to those inferred by SCINA or Garnett (columns). The color represents the recall and precision score (as a percent) of each original cell type predicted by SCINA or Garnett. Recall and precision scores no less than 20 are labelled. (A) Annotation of SCINA at the second layer 2 using optimized gene sets. Annotation of Garnett using one-step train(B) and hierarchical train(C) in layer 2.


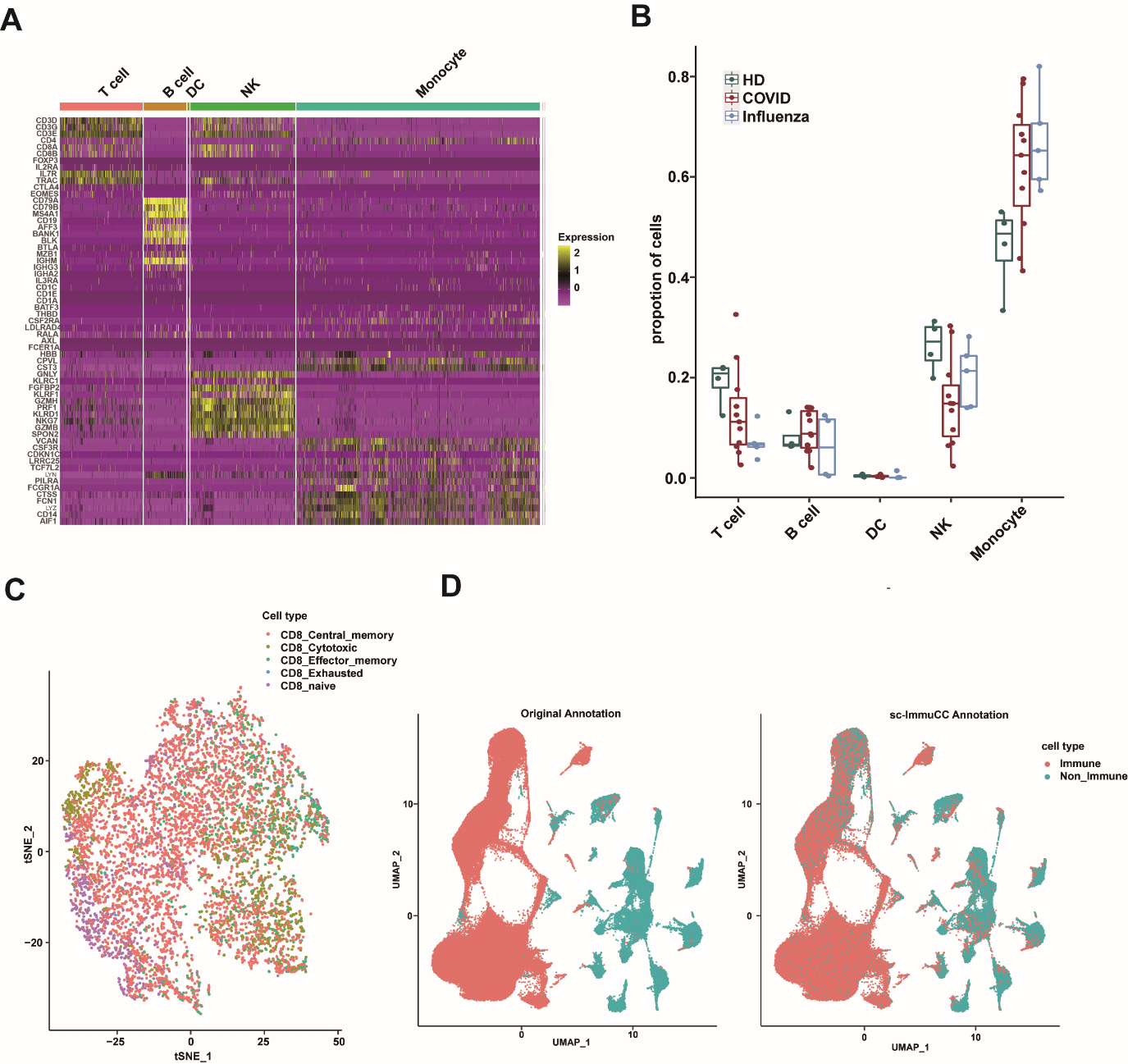


**Supplemental Figure 5.** Application of sc-ImmuCC. (A) Gene expression heatmap of sc-ImmuCC relabeled GSE149689 dataset at the first layer. (B) The proportion of immune cells at the first layer between the disease group and the healthy group. (C) tSNE plot of all third layer CD8 T cell subtypes in the dataset. (D) sc-ImmuCC can distinguish immune cells and non-immune cells. The left is the original annotated, and the right is annotated by sc-ImmuCC.
